# Vitamin B6 Deficiency Induces Autism-Like Behaviors in Rats by Regulating mTOR-Mediated Autophagy in the Hippocampus

**DOI:** 10.1155/2023/6991826

**Published:** 2023-05-09

**Authors:** Lijuan Chen, Jing Li, Xinglian Liu, Zhiwei Zhao, Yan Jin, Yikun Fu, Aiqin Zhou, Chengqun Wang, Yan Zhou

**Affiliations:** ^1^Department of Pediatric Neurology, Hubei Maternal and Child Health Hospital, Wuhan, China; ^2^Children's Health Department, Hubei Maternal and Child Health Hospital, Wuhan, China; ^3^Mass Spectrometry Center, Wuhan KingMed Diagnostics Group Co., Ltd, Wuhan, China

## Abstract

Vitamin B6 (VB_6_) exhibits therapeutic effects towards autism spectrum disorder (ASD), but its specific mechanism is poorly understood. Rat dams were treated with VB_6_ standard, VB_6_ deficiency, or VB_6_ supplementary diet, and the same treatment was provided to their offspring, with their body weights monitored. Three-chambered social test and open field test were employed to evaluate the effect of VB_6_ on autism-like behaviors. Gamma-aminobutyric acid (GABA) generation and synaptic inhibition of neurons in the hippocampus of rat were detected via immunofluorescence staining, followed by the measurement of GABA concentration through high-performance liquid chromatography (HPLC). The role of VB_6_ in the autophagy and apoptosis of cells was determined via Western blot and terminal deoxynucleotidyl transferase dUTP nick-end labeling (TUNEL). In order to conduct rescue experiments, the inhibition of mammalian target of rapamycin (mTOR) or the activation of GABA was achieved by drug administration to the offspring rats with VB_6_ deficiency. As a result, no evident difference in weight was observed in the offspring with varied VB_6_ treatments. VB_6_ deficiency impaired social interaction; aggravated self-grooming and bowel frequency; decreased GABA concentration, VIAAT, GAD67, vGAT expressions, and LC3 II/LC3 I ratio; increased p62 level and p-mTOR/mTOR ratio; and promoted cell apoptosis. Inhibition of mTOR reversed the effect of VB_6_ deficiency on cell autophagy. GABA activation or mTOR inhibition offset the role of VB_6_ deficiency in autism-like behaviors and hippocampal GABA expression. Collectively, VB_6_ deficiency induces autism-like behaviors in rats by regulating mTOR-mediated autophagy in the hippocampus.

## 1. Introduction

Autism spectrum disorder (ASD) is a pervasive neurodevelopmental disease with core symptoms of impaired social interaction, repetitive stereotypical behaviors, and narrow interests [1–3]. Unfortunately, there is no effective treatment for ASD, and children with this disease have poor social adaptability, bringing great emotional and economic burdens to society and families, which, therefore, has aroused widespread attention. It is believed that ASD is caused by a combination of both polygenic genetic and external environmental factors. Pathogenetically, during early embryonic development, patients with ASD are affected by a variety of factors that impair neuronal function and gamma-aminobutyric acid (GABA)ergic synaptic transmission, which in turn influence the balance of excitatory/inhibitory signal ratio, leading to autism-like behaviors [4]. Interneuronal GABAergic disorders have been observed in many animal models of ASD. Thus, repairing the dysfunction of the GABA system is thought to be an important way to alleviate ASD [5].

As an essential nutrient for the body, vitamin makes a profound impact upon the development and function of the brain and nervous system [6]. In recent years, multiple vitamins have been proved to alleviate ASD, including vitamin A (VA), VB_6_, and VD [7, 8]. VB_6_, a pyridine derivative, works as a coenzyme factor in diverse metabolic reactions of the human body, including amino acid metabolism, synthesis and breakdown of neurotransmitters, regulation of steroid hormone activity, and control of gene expression [9, 10]. Although the combination therapy using VB_6_ has been reported to be applied for ASD at present [11], knowledge gaps on its specific mechanism remain. However, existing research suggests that it might be associated with the ability of VB_6_ to strengthen the neurotransmitter system [12, 13].

Autophagy is a process of transporting misfolded proteins or damaged cell organelles, dysfunctional mitochondria, for instance, through autophagic vesicles to lysosomes for degradation [14]. Autophagy is a pivotal mechanism for cells to maintain homeostasis, the imbalance of which in neurons is usually linked to cerebral diseases, including ASD and neurodegenerative diseases [15–18]. Previous studies have pointed out that disruption of mammalian target of rapamycin (mTOR)-regulated macroautophagy and autophagy leads to autism-like abnormalities [19, 20]. Recently, it has been additionally demonstrated that enhancing mTOR-mediated autophagy contributes to the restoration of GABAergic signaling [21], which might alleviate autism-like behaviors in rats [22].

Three regions of the adult brain, the olfactory bulb, hypothalamus, and hippocampal dentate gyrus, contain newborn neurons that are crucial players in the natural functional circuits [23]. Many neurodegenerative diseases and neurodevelopmental disorders involving cognitive impairment may be associated with hippocampal dysfunction, which is at least partially attributable to adult neurogenetic disorders [24]. A recent review stated that hippocampus may represent an important component within a system of changed brain regions that work in tandem to contribute to the phenotype of ASD [25]. Moreover, VA deficiency induces autistic-like behaviors in rats through modulating the RAR*β*-CD38-oxytocin axis in the hypothalamus [26]. Thus, the hippocampus was chosen for the study of VB_6_ in ASD.

In this study, to suss out the role of VB_6_ in ASD, we firstly constructed the models in the offspring rat with VB_6_ deficiency or VB_6_ supplement and then explored whether the mechanism of VB_6_ in ASD was associated with mTOR-mediated autophagy, with the aim to provide new insight into the pathogenesis of ASD.

## 2. Material and Methods

### 2.1. Animals

The surgical procedures with animals in this study were approved by the Committee of Zhejiang Baiyue Biotech Co., Ltd for Experimental Animals Welfare (approval number: ZJBYLA-IACUC-20220701) and performed in accordance with Guide for the Care and Use of Laboratory Animals. A total of 24 virgin female Wistar rats (120–135 g) were purchased from Charles River Labs (Beijing, China) and were group-housed in standard animal cages (25 ± 2°C) with consistent 12/12 hours light/dark cycle.

### 2.2. Modeling

Autistic rat models with VB_6_ deficiency or VB_6_ supplement were constructed using the offspring of female Wistar rats as previously described [12]. Based on a basic diet, all female rats were randomly fed with 0, 6, or 30 mg VB_6_ (P5669, Sigma-Aldrich, St. Louis, MO, USA) for 4 weeks. During the pregnancy and lactation, in consistent with the grouping of their mother rats, the rats were continued to be given aforementioned VB_6_ treatments. After weaning, new pups (male) were collected and divided into the three groups: Control group (*n* = 6, a basic diet with 6 mg VB_6_), VB_6_ deficiency group (*n* = 24, a basic diet without VB_6_), and VB_6_ supplement group (*n* = 6, a basic diet with 30 mg VB_6_). The body weight of offspring rats was recorded from postnatal day 1 (PND 1) to PND 42. The offspring rats between PND 42 and PND 56 were subjected to the behavioral tests. The mother rats were sacrificed by cervical dislocation under anesthesia (40 mg/kg, pentobarbital sodium, P-010, Sigma-Aldrich, USA).

### 2.3. Drug Administration

GABA receptor agonist Clonazepam (C-907, Sigma-Aldrich, USA) and mTOR inhibitor NVP-BEZ235 (A10133, Adooq Bioscience, China) were used for drug administration *in vivo*. A total of 18 offspring rats were randomly selected from the abovementioned VB_6_ deficiency group and assigned into a new VB_6_ deficiency group (a basic diet without VB_6_), VB_6_ deficiency + Clonazepam group (intraperitoneal injection of 0.025 mg/kg Clonazepam based on the VB_6_ deficiency diet), and VB_6_ deficiency + NVP-BEZ235 group (intraperitoneal injection of 400 *μ*g/kg NVP-BEZ235 based on the VB_6_ deficiency diet), with six rats in each group. All offspring rats in this experiment were reared for 42–56 days since PND 1, and the drug administration was implemented 30 minutes prior to the behavioral tests [20, 22].

### 2.4. Behavioral Tests

#### 2.4.1. Three-Chambered Social Test

A three-chambered apparatus (XR-XJ117) purchased from XinRuan Information Technology (Shanghai, China) was applied to conduct sociability test [22]. After 10-minute habituation, the offspring rats (PND 42 and PND 56) with or without the indicated treatment of VB_6_ or drugs above were individually placed into the center chamber of the apparatus and allowed to access three chambers for 10 minutes, with an identical cage in each side of the chamber, one of which contained an unacquainted rat. The time the offspring rats spent in each chamber was automatically recorded.

#### 2.4.2. Open Field Test

The locomotor activity of the offspring rats (PND 42 and PND 56) with or without the indicated treatment of VB_6_ or drugs above was evaluated via the open field test with the assistance of an uncovered box (100 × 100 × 50 cm, XR-XZ301, XinRuan Information Technology, China). The box was cleaned thoroughly and sterilized with 75% alcohol (E299578, Aladdin, China) before the tests to eliminate any odors. The open field test was carried out according to the previous instructions [26]. Briefly, the offspring rats were individually placed into the center of the open field and allowed to explore the new environment without any disturbance for 15 minutes during the test. The SuperMaze software (vision 2.0, XR-Xmaze, XinRuan Information Technology, China) was applied to monitor movement speed, self-grooming time, and bowel frequency automatically during the test.

After behavioral test, all offspring rats were sacrificed by cervical dislocation under anesthesia, and the hippocampal tissues were harvested and stored in liquid nitrogen at −80°C for follow-up experiments. The right hemisphere was used for immunocytochemistry and terminal deoxynucleotidyl transferase dUTP nick-end labeling (TUNEL) experiments, while the hippocampus from the other hemisphere was utilized for Western blot. The hippocampal tissues from both hemispheres were employed for high-performance liquid chromatography (HPLC) assay. The experimental design was described in Supplementary Figure 1.

### 2.5. Immunofluorescence Analysis

The frozen hippocampal tissues were cut into sections with a thickness of 5 *μ*m and fixed with 4% paraformaldehyde (P0099, Beyotime, China) for 15 minutes. After being washed with phosphate buffered saline (PBS, P1022, Solarbio, China), the sections were subjected to 15 minute treatment with Triton X-100 (T8200, Solarbio, China) and 1 hour blocking using 5% goat serum (abs933, Absin, China). To determine the expressions of vesicular inhibitory amino acid transporter (VIAAT) and glutamate decarboxylase 67 (GAD67) in hippocampal CA3 area, the sections were incubated with primary antibodies against VIAAT (1 : 1000, ab101934, Abcam, Cambridge, UK) and GAD67 (10 *μ*g/ml, ab26116, Abcam, UK) at 4°C overnight away from light. Then, the sections underwent 1 hour incubation with secondary antibodies Alexa Fluor 488-labeled Goat Anti-Rabbit IgG (1 : 1000, P0176, Beyotime, China) and Alexa Fluor 647-labeled Goat Anti-Mouse IgG (1 : 1000, P0191, Beyotime, China) at room temperature. After 5 minute counterstain with 2-(4-amidinophenyl)-6-indolecarbamidine dihydrochloride (DAPI, C1005, Beyotime, China), fluorescent images were captured using a confocal microscope (LSM 780, ZEISS, Germany) at ×600 magnification.

To detect colocalization between GAD67 positivity (GAD67^+^) with vesicular gamma-aminobutyric acid transporter (vGAT) in hippocampal CA3 area, the sections were pre-treated with Triton X-100 and 5% goat serum as above and then were cultivated with primary antibodies against GAD67 and vGAT (1 : 500, 131006, Synaptic Systems, Germany) at 4°C overnight in the dark. The next day, the sections were cultured with secondary antibodies Alexa Fluor 488-Labeled Goat anti-Mouse IgG (1 : 1000, P0188, Beyotime, China) and Alexa Fluor 680-labeled Donkey Anti-Chicken IgG (1 : 100, 703-625-155, Jackson Immuno Research, USA), followed by DAPI staining. Lastly, a confocal microscope was used to observe the stained sections under ×400 magnification.

For investigating the activity of GABA^+^ in hippocampal CA3 area from hemispheres of VB_6_-deficient offspring rats with or without drug administration, the pre-treated sections were cultured with primary antibody targeting GABA (1 : 2000, ab8891, Abcam, UK) at 4°C overnight without light, followed by the cultivation with Alexa Fluor 647-labeled Goat Anti-Rabbit IgG Secondary Antibody (P0180, Beyotime, China) at room temperature for 1 hour. After the staining with DAPI, fluorescent images were obtained by a microscope at ×100 magnification. A total of three hippocampal hemispheres from six consecutive serial sections were used.

### 2.6. HPLC Assay

The content of GABA in the hippocampi of rats from Control, VB_6_ deficiency, and VB_6_ supplement groups was measured by HPLC assay. In brief, the hippocampal tissues from both hemispheres were homogenized in perchlorate solution (0.04 M, abs47048087, Absin, China) on ice, subsequent to which 15-minute centrifugation was carried out. Then, the supernatants were neutralized with potassium bicarbonate solution (2 M, abs42025446, Absin, China). After centrifugation, 2 ml supernatant of the sample or standard GABA solution was treated with triethylamine-acetonitrile (1 ml, 1/10, v/v) and phenyl isothiocyanate (PITC) acetonitrile (1 ml, 1/25, v/v) at room temperature for 1 hour away from light, followed by 20 minute *n*-hexane treatment (2 ml, H100107, Aladdin, China). Following the filtration, the lower layer solution (1 ml) was harvested for analysis. HPLC was conducted under an Agilent 1100 HPLC system (Agilent, Santa Clara, CA, USA) to analyze the content of GABA according to the previous protocols [22]. Assay for each sample was repeated twice.

### 2.7. Western Blot

The levels of autophagy-related proteins in the brain tissues from rats in VB_6_ deficiency group with or without drug administration, VB_6_ supplement group, and Control group were measured via Western blot. After the extraction of total protein with RIPA Lysis Buffer (E-BC-R327, Elabscience, China), BCA Protein Assay Kit (23225, ThermoFisher, Waltham, MA, USA) was used to detect protein concentration in the collected supernatant following the instructions. Afterwards, equal amount of protein (50 *μ*g/lane) was separated by 10% sodium dodecyl sulfate-polyacrylamide gel electrophoresis (SDS-PAGE, E-IR-R305, Elabscience, China) and loaded onto nitrocellulose membranes (2215, Millipore, Billerica, MA, USA). Next, the membranes were sequentially treated with blocking buffer at room temperature for 1 hour and cultivated with primary antibodies at 4°C overnight. The next day, the membranes were rinsed with tris-buffered saline with Tween-20 (TBS-T, abs952, Absin, China) and incubated with secondary antibody at room temperature for 2 hours. For the visualization of immunoblots, the protein bands were added with PLUS Chemiluminescent Substrate (34577, ThermoFisher, USA) and developed in a Tanon 5200 Imaging System (Shanghai, China). All antibodies were obtained from Abcam (UK), and the primary antibodies were those against LC3 I/LC3 II (1/1000, 14 kDa, 16 kDa, ab192890), p62 (1/10000, 62 kDa, ab109012), mTOR (1/1000, 250 kDa, ab32028), phosphor (p)-mTOR (1/1000, 289 kDa, ab109268), and glyceraldehyde-3-phosphate dehydrogenase (GAPDH, 0.1 *μ*g/ml, 36 kDa, ab9484). The secondary antibodies consisted of Rabbit Anti-Mouse IgG (1/2000, ab6728) and Goat Anti-Rabbit IgG (1/3000, ab6721).

### 2.8. TUNEL Assay

Cell apoptosis in CA1 and CA3 areas of rat hippocampus from Control, VB_6_ deficiency, and VB_6_ supplement groups was determined using Colorimetric TUNEL Apoptosis Assay Kit (C1091, Beyotime, China). The frozen hippocampal sections were treated with Immunol Staining Fix Solution (P0098, Beyotime, China) at room temperature for 30 minutes, followed by the rinse using PBS twice. Then, each section was immersed into Enhanced Immunostaining Permeabilization Buffer (P0097, Beyotime, China) for 5 minutes, blocked with 0.3% H_2_O_2_ in PBS for 20 minutes, and reacted with 50 *μ*l prepared biotin-labeled solution at 37°C in a wet box for 1 hour without light. After the reaction was terminated, the sections were subjected to color development, including the treatment of both streptavidin–horseradish peroxidase (HRP) (50 *μ*l) and 3,3′-diaminobenzidine (DAB, 0.2 ml) according to the manufacturer's specification. Following dehydration with gradient alcohol and hyalinization with xylene, the number of positively apoptotic cells in the hippocampal CA1 and CA3 by cells/0.01 mm^2^ was counted using a light microscope (×100 magnification, CX23, Olympus, Japan). Three hippocampal hemispheres from six consecutive serial sections were used.

### 2.9. Statistical Analysis

The results of triplicate assays were expressed as mean ± standard deviation. Kolmogorov–Smirnov test was used to verify normality. One-way analysis of variance was used for multi-group comparison, followed by Tukey or Dunnett's *post-hoc* test to detect any inter-group differences. Statistical analyses were realized using Graphpad Prism 8.0 software (GraphPad Software Inc., San Diego, CA, USA). *P* < 0.05 indicated the data were statistically significant.

## 3. Results

### 3.1. VB_6_ Deficiency Reduced Sociability and Aggravated Anxiety-Related Behaviors of Offspring Rats

**Figure 1:**
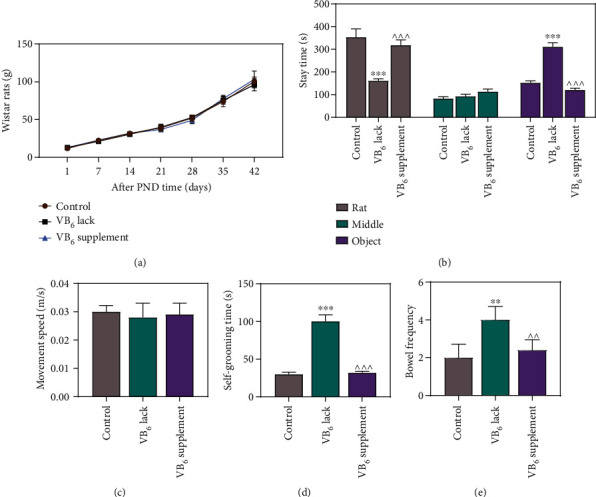
VB_6_ deficiency reduced sociability and aggravated anxiety-related behaviors of offspring rats. The offspring rats obtained from pregnant rats with VB_6_ normality, VB_6_ deficiency, or VB_6_ supplement were subjected to the modeling. (a) The body weight of offspring rats was recorded from PND 1 to PND 42. (b) Three-chambered social test was utilized to evaluate the social interactions of offspring rats. (c, d, and e) The movement speed, self-grooming time, and bowel frequency of offspring rats were detected by open field test. Six rats/group. One-way analysis of variance with Tukey's *post-hoc* test. VB_6_: vitamin B6; PND: postnatal day; ∗∗*p* < 0.01; ∗∗∗*p* < 0.001 vs. Control; ^^*p* < 0.01; ^^^*p* < 0.001 vs. VB_6_ lack.

No abnormal deaths or loss were observed in all rats during this study. To explore the role of VB_6_ in rats with autism-like behaviors, we obtained the offspring from pregnant rats to construct rat model with VB_6_ normality, VB_6_ deficiency, or VB_6_ supplement. As exhibited in weight curves (Figure 1(a)), no obvious difference was reported concerning the weight of the offspring rats among the control, VB_6_ deficiency, and VB_6_ supplement groups. Subsequently, we investigated whether VB_6_ has an effect on autism-like behaviors of rats. The evaluation of social interactions demonstrated that when compared to the control group, in VB_6_ deficiency group, the offspring rats had fewer social interactions with unfamiliar rats but more interactions with the object, and in VB_6_ supplement group, the offspring rats had more social interactions with unfamiliar rats and less interactions with the object (Figure 1(b), *p* < 0.05). In line with the open field test results, we observed that VB_6_ deficiency and VB_6_ supplement exerted no effect on movement speed of the offspring rats (Figure 1(c)), while VB_6_ deficiency increased self-grooming time and bowel frequency (Figures 1(d) and 1(e), *p* < 0.01).

### 3.2. VB_6_ Deficiency Diminished Synaptic Inhibition in the Hippocampi of Rats

**Figure 2:**
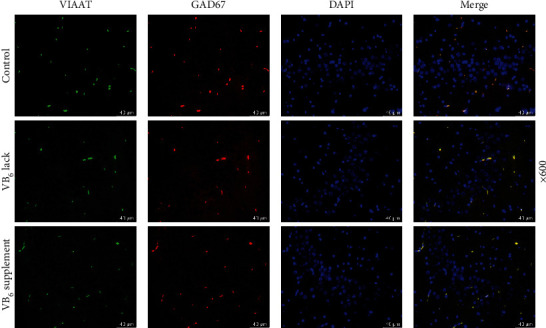
VB_6_ deficiency reduced GABAergic inhibition in hippocampal CA3 area of offspring rats. The offspring rats obtained from their pregnant mothers with VB_6_ normality, VB_6_ deficiency, or VB_6_ supplement were modeled to evaluate autism-like behaviors. Hippocampal tissues were collected after euthanasia of offspring rats. The expressions of VIAAT and GAD67 in the hippocampal CA3 area of rat were determined using immunofluorescence staining (×600 magnification, scale bar: 40 *μ*m). Three rats/group. One-way analysis of variance with Tukey's *post-hoc* test. VB_6_: vitamin B6; VIAAT: vesicular inhibitory amino acid transporter; GAD67: glutamate decarboxylase 67.

**Figure 3:**
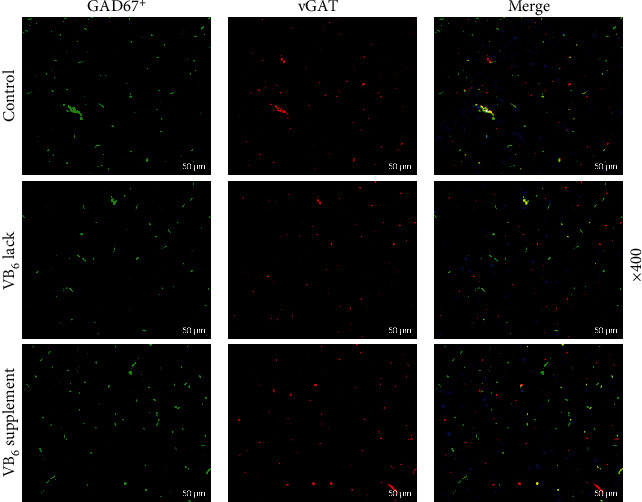
VB_6_ deficiency diminished GAD67^+^ inhibitory synapses in hippocampal CA3 area of offspring rats. The offspring rats obtained from pregnant mothers with VB_6_ normality, VB_6_ deficiency, or VB_6_ supplement were modeled. Hippocampal tissues were collected after euthanasia. Immunofluorescence analysis was used to detect synaptic inhibition of neurons in the hippocampus by staining GAD67^+^ and vGAT (×400 magnification, scale bar: 50 *μ*m). Three rats/group. One-way analysis of variance with Tukey's *post-hoc* test. VB_6_: vitamin B6; GAD67^+^: glutamate decarboxylase 67-positive; vGAT: vesicular gamma-aminobutyric acid transporter.

**Figure 4:**
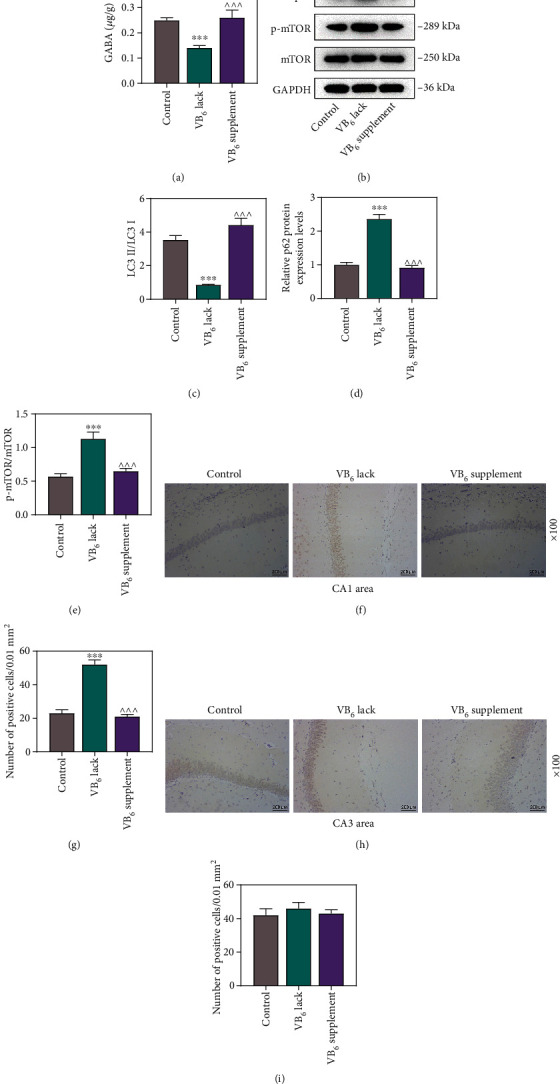
VB_6_ deficiency suppressed GABA content and autophagy and accelerated apoptosis of neurons in hippocampus of offspring rats. The offspring rats obtained from pregnant mothers with VB_6_ normality, VB_6_ deficiency, or VB_6_ supplement were constructed to evaluate autism-like behaviors. Hippocampal tissues were collected after sacrifice of offspring rats. (a) The concentration of GABA in rat hippocampus was measured by high-performance liquid chromatography. (b, c, d, and e) Western blot was applied to measure the levels of LC3 I, LC3 II, p62, mTOR, and p-mTOR proteins in the hippocampal tissues. GAPDH was used as the loading control. (f, g, h, and i) The number of apoptotic cells in rat hippocampal CA1 and CA3 areas was determined using TUNEL assay (×100 magnification, scale bar: 200 *μ*m). Three rats/group. One-way analysis of variance with Tukey's *post-hoc* test. VB_6_: vitamin B6; GABA: gamma-aminobutyric acid; mTOR: mammalian target of rapamycin; p-mTOR: phosphor-mTOR; GAPDH: glyceraldehyde-3-phosphate dehydrogenase; TUNEL: TdT-mediated dUTP nick-end labeling; ∗∗∗*p* < 0.001 vs. Control; ^^^*p* < 0.001 vs. VB_6_ lack.

The immunofluorescence analysis was conducted to reveal the effect of VB_6_ on synaptic inhibition of neurons in the hippocampus of offspring rats. As illustrated in Figure 2, the density of VIAAT and GAD67 fluorescence signals was lessened in hippocampal CA3 area of the offspring rats with VB_6_ deficiency, and no remarkable change was observed in the offspring rats with VB_6_ supplement. Additionally, the hippocampal CA3 area of offspring rats with VB_6_ deficiency exhibited conspicuously decreased GAD67^+^ and vGAT, while no significant change was visible in the offspring rats with VB_6_ supplement (Figure 3). Moreover, the results of HPLC assay revealed that the concentration of GABA was dwindled in the hippocampus of offspring rats in VB_6_ deficiency group, compared to that in Control group (Figure 4(a), *p* < 0.001).

### 3.3. VB_6_ Deficiency Suppressed Autophagy and Accelerated Apoptosis of Neurons in the Offspring Rats

Considering that the imbalance of autophagy is closely associated with cerebral diseases including autism [15], Western blot was employed for measuring the protein levels of LC3 I, LC3 II, p62, mTOR, and p-mTOR in the hippocampal tissues. As compared with those in Control group, decreased LC3 II/LC3 I ratio and increased p-mTOR/mTOR ratio and p62 protein level were observed in the offspring rats in VB_6_ deficiency group, while increased LC3 II/LC3 I ratio was observed in the offspring rats in VB_6_ supplement group (Figures 4(b), 4(c), 4(d), and 4(e), *p* < 0.001). We also detected cell apoptosis in hippocampal CA1 and CA3 areas using TUNEL assay. Notably, more cell apoptosis in the hippocampal CA1 area emerged in VB_6_ deficiency group, while less apoptotic cells appeared in VB_6_ supplement group (Figures 4(f) and 4(g), *p* < 0.001). In addition, VB_6_ deficiency or VB_6_ supplement generated no significant effect on cell apoptosis in the hippocampal CA3 area (Figures 4(h) and 4(i)).

### 3.4. The Role of VB_6_ in the Autism-Like Behaviors of Rats Was Mediated by mTOR Inhibition and GABA Activation

**Figure 5:**
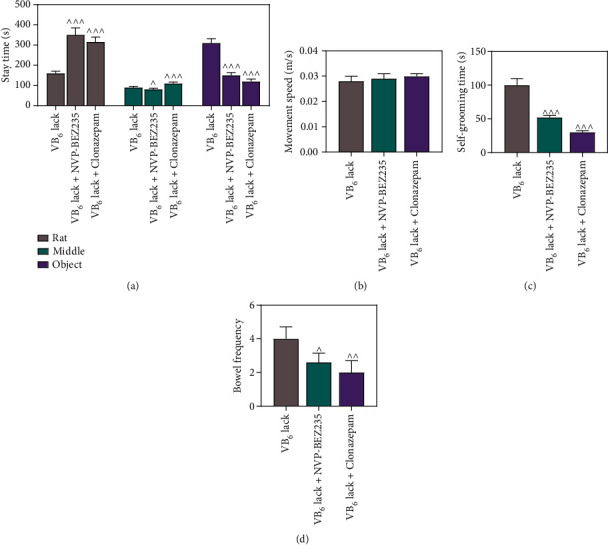
VB_6_ deficiency induced autism-like behaviors by inhibiting the generation of GABAergic interneurons or activating mTOR pathway in the hippocampus of offspring rats. The modeling of autism-like behaviors was constructed in offspring rats obtained from pregnant mothers with VB_6_ deficiency, and the offspring was intraperitoneally injected with NVP-BEZ235 (400 *μ*g/kg) or Clonazepam (0.025 mg/kg) 30 minutes before behavioral tests. (a) After drug administration, three-chambered social test was utilized to evaluate the social interactions of offspring rats. (b, c, and d) The movement speed, self-grooming time, and bowel frequency of offspring rats were detected by open field test. Six rats/group. One-way analysis of variance with Dunnett's *post-hoc* test. VB_6_: vitamin B6; ^*p* < 0.05; ^^*p* < 0.01; ^^^*p* < 0.001 vs. VB_6_ deficiency.

**Figure 6:**
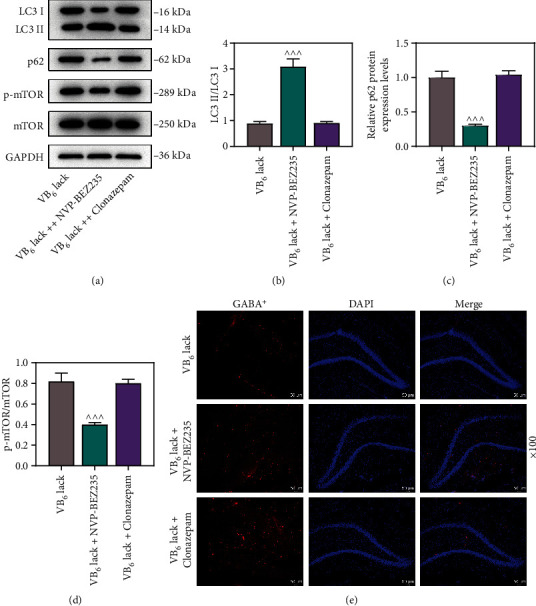
VB_6_ deficiency suppressed generation of GABAergic interneurons and autophagy by activating mTOR pathway in hippocampus in offspring rats. The offspring rats obtained from their pregnant mothers with VB_6_ deficiency was subjected to the modeling and were intraperitoneally injected with NVP-BEZ235 (400 *μ*g/kg) or Clonazepam (0.025 mg/kg) 30 minutes before behavioral tests. Following drug administration, hippocampal tissues were collected after euthanasia of offspring. (a, b, c, and d) The levels of autophagy-related proteins (LC3 I, LC3 II, and p62) and mTOR pathway-related proteins (mTOR and p-mTOR) were measured by Western blot. GAPDH was used as the loading control. (e) The expression of GABA^+^ in the hippocampal CA3 area of offspring rats was determined using immunofluorescence staining (×100 magnification, scale bar: 50 *μ*m). Three rats/group. One-way analysis of variance with Dunnett's *post-hoc* test. VB_6_: vitamin B6; GABA^+^: gamma-aminobutyric acid-positive; mTOR: mammalian target of rapamycin; p-mTOR: phosphor-mTOR; GAPDH: glyceraldehyde-3-phosphate dehydrogenase; ^^^*p* < 0.001 vs. VB_6_ deficiency.

It has been suggested that the role of mTOR-mediated autophagy in the development of autism might be correlated with GABA [22]. Hence, we triggered mTOR inhibition or GABA activation in the offspring rats with VB_6_ deficiency by drug administration with NVP-BEZ235 or Clonazepam and conducted rescue assays. The results of three-chambered social test unveiled that both NVP-BEZ235 and Clonazepam promoted social interaction of the offspring rats with VB_6_ deficiency, compared to those without drug administration (Figure 5(a), *p* < 0.001). As demonstrated in Figures 5(b), 5(c), and 5(d), neither NVP-BEZ235 nor Clonazepam signally influenced the movement speed, and VB_6_ deficiency-induced increments in self-grooming time and bowel frequency were dwindled by both NVP-BEZ235 and Clonazepam (*p* < 0.05). At molecular level, NVP-BEZ235 increased LC3 II/LC3 I ratio, decreased p-mTOR/mTOR ratio, and down-regulated p62 protein level, which reversed the effects of VB_6_ deficiency (Figures 6(a), 6(b), 6(c), and 6(d), *p* < 0.001). No observable effect of Clonazepam, however, was detected on LC3 II/LC3 I ratio, p-mTOR/mTOR ratio, and p62 protein level (Figures 6(a), 6(b), 6(c), and 6(d)). Interestingly, the results of immunofluorescence staining demonstrated that both NVP-BEZ235 and Clonazepam enhanced GABA^+^ in the hippocampi of the offspring rats with VB_6_ deficiency, compared to those without drug administration (Figure 6(e)). Finally, a scheme showing the roles of VB_6_, GABA transmission, mTOR, and apoptosis in the proposed mechanism of the autism etiology was presented in Supplementary Figure 2.

## 4. Discussion

The treatment for ASD using VB_6_ could date back to the 1960s, and years of clinical studies have proven that oral administration with high-dose VB_6_ can significantly alleviate autism-like behaviors in patients [27]. Persistent stereotypical and repetitive behaviors, narrow interests, and lagging social skills constitute the main symptoms of ASD [28]. Through the behavioral tests in animal, we observed a notable lack of social interest and strong repetitive stereotypic behaviors in the VB_6_-deficient model rats, but the deficiency and supplementation of VB_6_ did not markedly affect body weight and locomotion in the model rats. It can be concluded that VB_6_ deficiency indeed participates in the development of ASD, anxiety, and cognitive behaviors in particular. Nevertheless, its regulatory mechanism in this process has not been fully understood.

Since VB_6_ can promote amino acid absorption and protein synthesis, catalyze the conversion of glutamate into GABA, and inhibit the central nervous system, it is often used clinically to treat epilepsy, isoniazid accumulation-induced central nervous system (CNS) excitation, and peripheral neuritis [29–31]. The deficiency of behavioral flexibility frequently observed in patients with ASD may be, at least in part, due to the inadequate control of GABAergic transmission by the regulatory system. GABA, produced by GABAergic inhibitory interneurons, is an important inhibitory transmitter in the CNS, and its down-regulation can cause dysfunction of central neurotransmitters, which results in delayed brain response, distraction, and emotional variability, thus affecting social interaction of patients with ASD [32–34]. The study of Almeida et al. indicated that the appearance of GABA neurotransmitter perturbations in the offspring may be attributed to maternal VB_6_ deficiency [12]. The above findings enlightened us that the repair of GABA system via VB_6_ could be a potential mechanism in alleviating ASD. Excitation/inhibition synaptic imbalance is considered as one of the most dominant neurophysiological features of ASD [35]. Synapses are specialized structures that connect and transmit between neurons or between neurons and effector cells, and their structural and quantitative integrity ensure the normal brain function. It has been shown that GABA is critical in regulating feedback and feedforward inhibition of neuronal excitability [36]. Therefore, we investigated the generation of GABA in the hippocampal CA3 area by immunofluorescence staining, and uncovered that the fluorescence density of GAD67, VIAAT as well as GAD67^+^, and VIAAT^+^ cells was prominently reduced in the neurons of VB_6_-deficient rat. Moreover, the results of HPLC assay verified that VB_6_ deficiency induced the diminution of GABA concentration in rat hippocampus. GAD67 is an isoform of GABA synthase that can catalyze the production of GABA from L-glutamate [37, 38]. VIAAT, as a marker of GABAergic presynaptic elements, is responsible for the uptake and transport of GABA from the synaptic gap to the synaptic vesicles to regulate the concentration of GABA in the synaptic gap and terminate inhibitory synaptic transmission. The expression level of VIAAT directly reflects the strength of synaptic transmission [39]. Our findings identified that VB_6_ deficiency impaired the production of GABA and reduces inhibitory synapses, thus enhancing the release of excitatory neurotransmitters from neurons, which may be an underlying mechanism of autism-like behaviors in rats.

LC3 is the most important marker during autophagy and is primarily involved in the formation of autophagosomes. p62 acts as a link between ubiquitinated proteins and LC3 to promote the degradation of autophagy. Numerous researches have revealed that the abnormally initiated autophagy-related pathways result in reduced autophagic activity, which is associated with the development of ASD [15, 40, 41]. Hui et al. unraveled that autophagy deficiency in the hippocampus contributes to the formation of SQSTM1/p62-positive aggregates, thereby compromise the transport function of GABA_A_ receptors [21]. mTOR is a critical downstream signaling molecule of adenosine 5′-monophosphate (AMP)-activated protein kinase (AMPK) and plays a negative regulatory role in the autophagy of cells. The research by Zhang et al. confirmed that autophagy acts as a link between mTOR and GABA signaling, and that mTOR inhibition promotes PI3K/AKT/mTOR-mediated autophagic activity, thereby enhancing social interaction in autistic rats [20]. As expected, we detected the elevated p62 protein level and the reduced LC3 II/LC3 I and p-mTOR/mTOR ratios in the hippocampal tissues of VB_6_-deficient rat and demonstrated that VB_6_ deficiency can inhibit the autophagy in rat hippocampus by the activation of mTOR, thereby reducing GABA generation and inhibitory synapses. Additionally, we verified that VB_6_ deficiency triggered cell apoptosis in the hippocampal CA1 area, which was in consistent with the study of Zhang et al. [20], proving that the decrease in inhibitory synapses was accompanied by an increase in the number of apoptotic interneurons in the hippocampus. In order to clarify the interaction between mTOR-mediated autophagy and GABA, we separately inhibited mTOR and activated GABA in VB_6_-deficent rat models by NVP-BEZ235 (mTOR inhibitor) and Clonazepam (which has been validated to enhance the function of GABA receptors) in animal models of ASD [42]. Based on the analysis of rescue experiments, we found that NVP-BEZ235 acted similarly to Clonazepam to improve autism-like behaviors and enhance hippocampal GABA^+^ expression in rats with VB_6_ deficiency, and meanwhile Clonazepam did not affect autophagic processes. Collectively, it was indicated that the inhibition of mTOR activated autophagy to down-regulate the protein level of p62, thus mitigating the dysfunction of GABA receptor caused by isolation of SQSTM1/p62-positive aggregates.

In conclusion, based on *in vivo* experiments, our current study authenticates that VB_6_ deficiency induces autism-like behaviors in rats by regulating mTOR-mediated autophagy, which provides a new mechanism of ASD. However, the absence of *in vitro* experiments is a shortcoming in this study.

## Data Availability

The analyzed data sets generated during the study are available from the corresponding author on reasonable request.

## Supplementary Materials

Supplementary Materials Supplemental Figures 1 and 2 are the experimental design and finding scheme (Supplementary Materials).
