# Supplementary material for: Vitamin B6 Deficiency Induces Autism-Like Behaviors in Rats by Regulating mTOR-Mediated Autophagy in the Hippocampus
Source: Behav Neurol. 2023 May 9;2023:6991826. doi: 10.1155/2023/6991826 (PMC10188270; doi:10.1155/2023/6991826)
Supplement: Supplementary Materials — Supplemental Figures 1 and 2 are the experimental design and finding scheme (Supplementary Materials). [file 6991826.f1.zip › Supplemental Figure 2.docx]

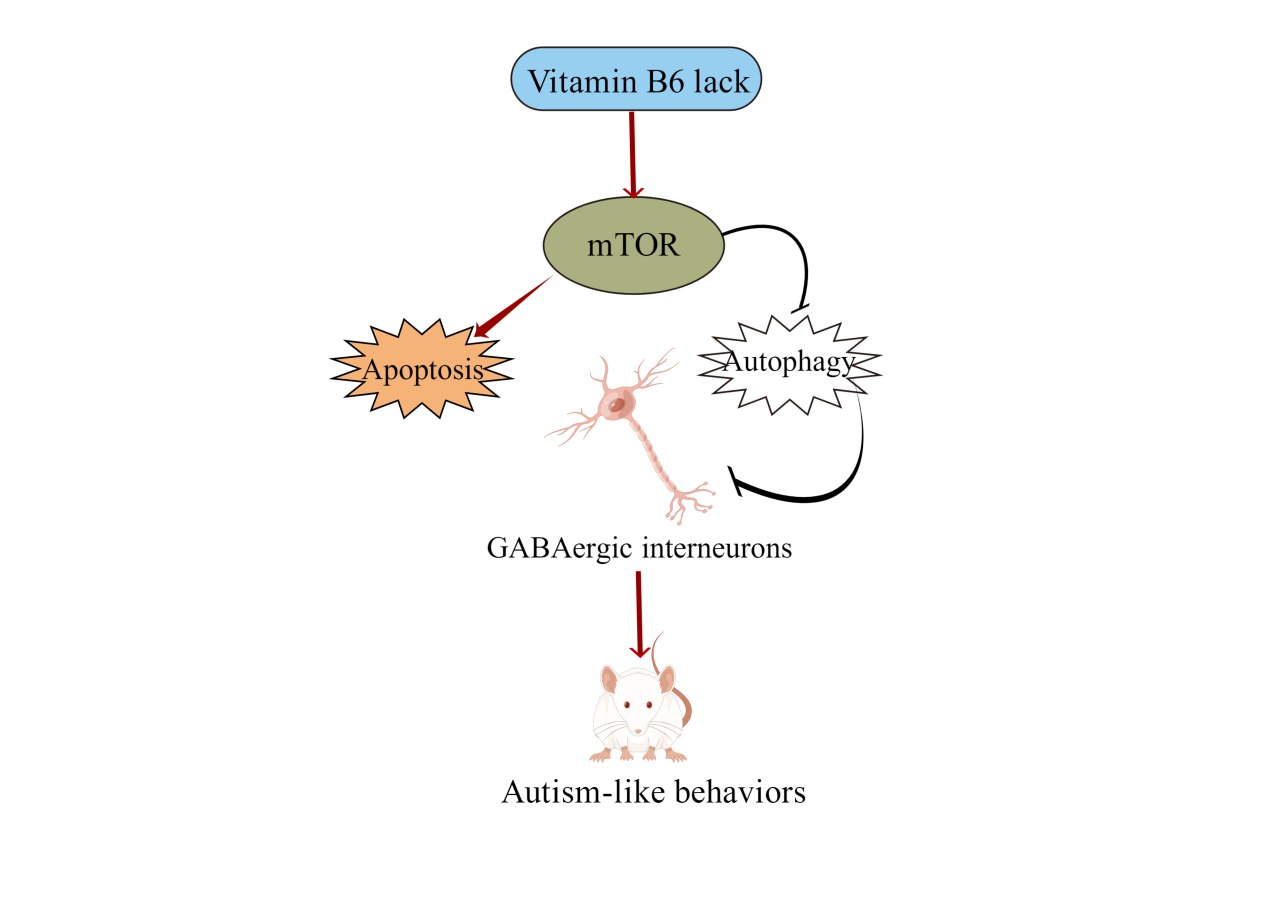


**Supplementary Figure 2.** A scheme showing the roles of Vitamin B6, GABA transmission, mTOR and apoptosis in the proposed mechanism of the autism etiology.
