# Supplementary figures and images for: Vitamin B6 Deficiency Induces Autism-Like Behaviors in Rats by Regulating mTOR-Mediated Autophagy in the Hippocampus
Source: Behav Neurol. 2023 May 9;2023:6991826. doi: 10.1155/2023/6991826 (PMC10188270; doi:10.1155/2023/6991826)

**
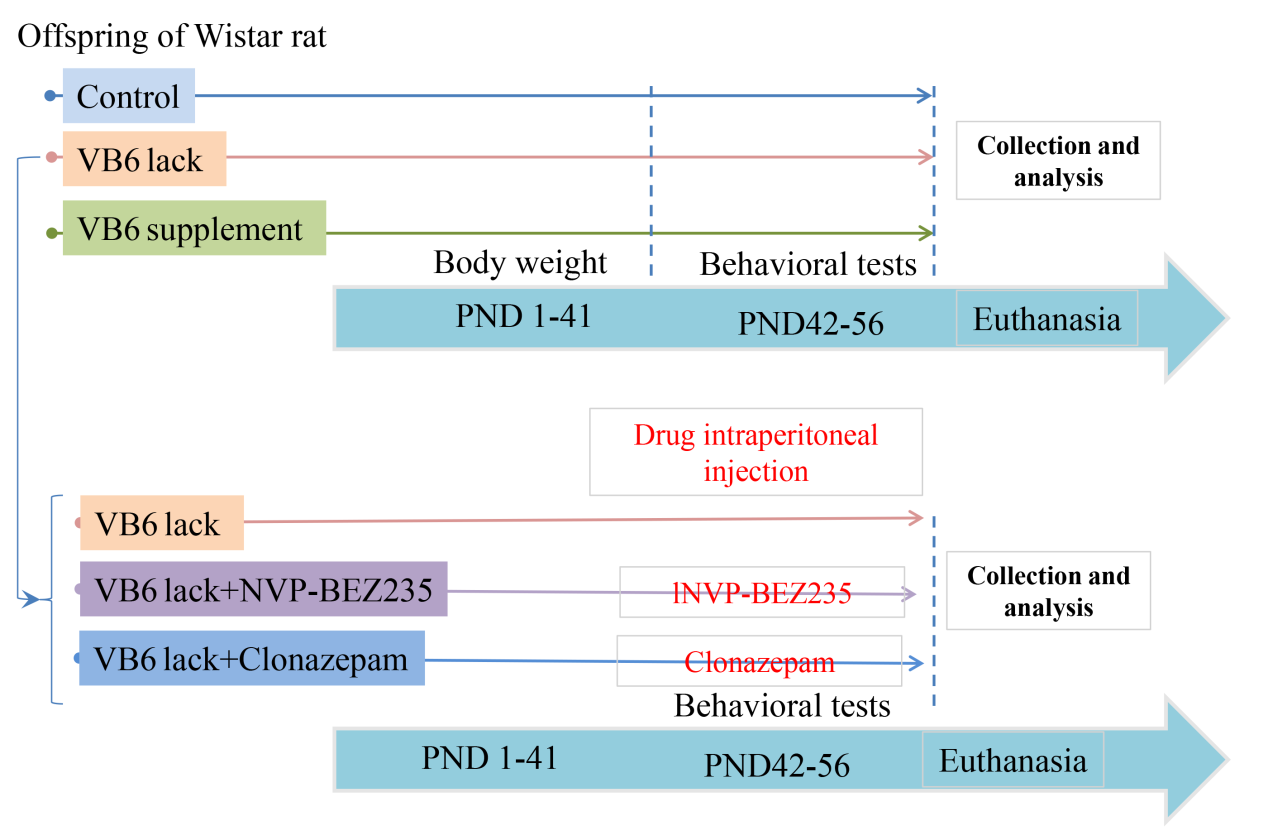
**

**Supplementary figure 1.** The experimental design.

Supplement: Supplementary Materials — Supplemental Figures 1 and 2 are the experimental design and finding scheme (Supplementary Materials). [file 6991826.f1.zip › Supplemental Figure 1.docx]
